# Hepatitis E seroprevalence and risk factors in humans and pig in Ghana

**DOI:** 10.1186/s12879-022-07127-6

**Published:** 2022-02-07

**Authors:** Husein Bagulo, Ayodele O. Majekodunmi, Susan C. Welburn, Langbong Bimi

**Affiliations:** 1 Zhejiang University – University of Edinburgh Institute, Zhejiang University International Campus, 718 East Haizhou Rd, Haining, Zhejiang 314400 People’s Republic of China; 2 Livestock and Poultry Research Centre, College of Basic and Applied Sciences, University of Ghana, Legon, P. O Box LG 38, Accra, Ghana; 3 Department of Animal Biology & Conservation Science, College of Basic and Applied Sciences, University of Ghana, Legon, Accra, Ghana; 4 School of Biomedical Sciences, Edinburgh Medical School, College of Medicine & Veterinary Medicine, The University of Edinburgh, 1 George Square, Edinburgh, EH8 9JZ UK

**Keywords:** Hepatitis E, Pigs, Water, Sanitation, Hygiene

## Abstract

**Background:**

Although Hepatitis E virus (HEV) infection has significant negative impact on the health and wellbeing of underprivileged populations, the burden of HEV in Ghana is still unclear, despite widespread water, sanitation, and hygiene (WASH) related conditions that predispose people to the risk of infection.

**Methods:**

A cross-sectional study was conducted to explore rates of HEV seroprevalence and HEV prevalence, as well as risk factors in humans and domestic pigs in Ghana. These were determined using ELISAs manufactured by Wantai Beijing Biopharmaceuticals, China. The study involved 1365 community members, 105 pig farmers and 474 domestic pigs from four administrative regions of Ghana.

**Results:**

Results showed overall seroprevalence and prevalence of 12.4% and 0.7% in community members and 15.2% and 2.9% in pig farmers respectively. There was no significant difference in the seroprevalence between the two groups (Z = 0.851; p = 0.3953). However, the prevalence in pig farmers was significantly higher than in community members (Z = 2.412; p = 0.016). Age (OR = 1.369, CI = 1.243 – 1.508; p = 0.0000), gender (OR = 1.419, CI = 1.101 – 1.991; p = 0.043), and the region of residence (OR = 1.569, CI = 1.348 – 1.827; p = 0.0000) were significant risk factors for HEV seroprevalence in a multivariate regression model.

In pigs, overall seroprevalence and prevalence of 62.4% and 5.5% were recorded respectively. A significant difference in seroprevalence was found between confined (6.7%) and free-range pigs (88.3%), (Z = 7.492; p < 0.00001) in the Volta Region. Multivariate logistic regression showed a significant association between seroprevalence and husbandry (OR = 7.051, CI = 3.558– 13.972; p = 0.0000) and region (OR = 4.602, CI = 2.300 – 9.205; p = 0.0000) in pigs in the Volta and Greater Accra Region.

**Conclusion:**

From this study, HEV is endemic in Ghana with high seroprevalence in humans and pig populations. This underscores the need for awareness creation and action for prevention and control.

## Introduction

Viral hepatitis present a major threat to public health security, causing severe morbidities and high mortalities through acute and chronic infections [1]. Hepatitis E virus (HEV) infection is recognised as one of the primary causes of acute viral hepatitis in humans worldwide [2]. It is a serious public health disease in many developing countries, especially in regions of high faecal contamination of drinking water supplies and poor sanitation [3]. As a neglected tropical disease related to water, sanitation, and hygiene (WASH), HEV infection has a huge burden on many underprivileged populations in developing countries but has gained insufficient attention in terms of control interventions, research, and treatment options [4, 5].

In Ghana, environmental risk factors for transmission of the infection to humans are ubiquitous: sanitation coverage is low at 15% with a 19% open defecation rate [6]. Also, there is widespread faecal contamination of drinking water [7]. Furthermore, domestic pigs, which are the primary host of the HEV are allowed to roam free in major pig production communities in Ghana; thus, serving as agents of environmental contamination by shedding the virus in faeces and urine.

The burden of this zoonotic infection is not well known in Ghana, although there have been considerable studies on the infection in the country. Results from some localised serosurveys in various populations groups in Ghana such as pig handlers or persons with contact with pigs [8–10], blood donors [11, 12], and pregnant women [13, 14] highlight high HEV seroprevalence but the prevalence (i.e. current infections) and risk of transmission to the general public are not yet known. While the research available provides some insight into the problem of HEV infection in Ghana, some knowledge gaps remain: Most of the previous studies featured small study areas and sample sizes and specific population groups rather than the general public. Also, very young children (age 1–5 years) were not involved.

To fill these gaps, a large cross-sectional study was conducted between October 2019 and October 2020 to investigate the contribution of zoonotic and WASH-related transmission routes to the burden of HEV infection in humans and domestic pigs in the South East of Ghana. HEV seroprevalence, prevalence and associated risk factors are presented in this paper. These results enable us to better gauge the regional distribution and drivers of HEV infection and the national burden of the disease, which are crucial for a better understanding of the epidemiology of the disease, improving awareness, and evidence-based decision making by policymakers in Ghana.

## Materials and methods

### Sampling frame and study sites

A pre-survey was carried out between July and September 2019 in all selected districts to confirm and sample communities with the desired characteristics. The district league tables of UNICEF and WaterAid Ghana were used as a guide to identify open defaecation and open defaecation-free communities. Contacts with prospective participants and local authorities were also made during the pre-survey.

The sampling frame included four [4] regions of Ghana, namely, the Greater Accra Region, the Volta Region, the Eastern Region, and the Central Region. In each region, two [2] districts were randomly selected and, in each district, two [2] communities were purposely selected targeting the desired community types giving a total of sixteen [16] communities.

Sanitation records from the Community-Led Total Sanitation programme [15] provided the open defaecation status of the study communities. A pre-survey provided data on other community characteristics.

**Fig. 1:**
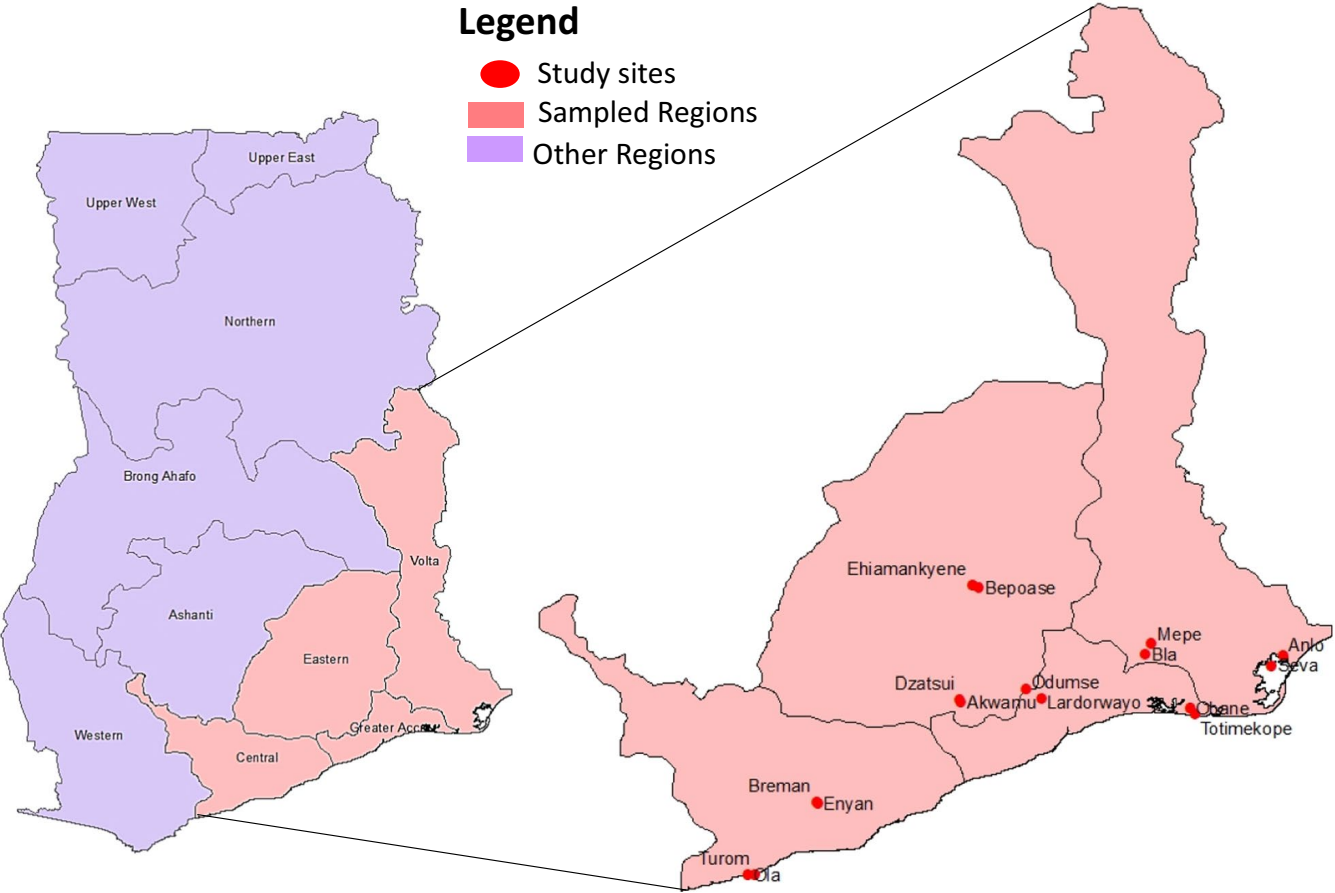
Study sites

The selected communities were a combination of ‘’presence or absence of open defaecation (OD)’’ and ‘’presence or absence of free-range pigs (PIG)’’ to explore the contribution of pigs and sanitation to the transmission of HEV. Thus, there were four categories of study communities: (1) Absence of both pigs and open defaecation (PIG− OD−), (2) Presence of both pigs and open defaecation (PIG + OD +), (3) Absence of pigs and presence of open defaecation (PIG− OD +), and (4) Presence of pigs and absence of open defaecation (PIG + OD−). The study sites are illustrated in Fig. 1 below.

### Study design and sample size estimation

A cross-sectional cluster survey method was employed for the study. CSurvey software version 2.0 was used to estimate sample sizes of 100 for humans and 160 for pigs per cluster at a desired level of precision of 0.05%. The estimation was done using HEV seroprevalence values of 37% and 85% for humans and pigs respectively determined from our previous study in Ghana [16]. Thus, a sample size of 100 people was allocated to each community consisting of 75 community members and 25 pig farmers (3:1 ratio). However, in communities without pig farmers only community members were sampled; thus, 100 community members. For pigs, since only two of the four communities sampled from each region included pigs, a sample size of 160 pigs was assigned to each community. Extensive sensitization exercise was carried during the pre-survey period and a day before sampling in the communities to ensure maximum participation in the study. However, in some communities, the desired sample sizes could not be achieved due to religious beliefs held by participants against blood sampling.

Table 1 shows the region, district, community, community status, and the number of research participants and pigs sampled from each study community.

**Table 1:** Number of participants and pigs sampled

Region	District	Community	Community Status	Community Members	Pig Farmers	Total Samples	Pigs
Accra	Ada East	Totimehkope	+ OD PIG−	99	6	105	30
		Obane	+ OD PIG +	43	15	58	89
	Shai Osudoku	Odumase	+ OD PIG +	91	3	94	83
		Lardor Wayo	−OD PIG +	80	4	84	-
Central	Ajumako	Turom	+ OD PIG +	90	4	94	-
		Ola Estate	+ OD PIG−	76	7	83	52
	Cape Coast Municipality	Breman Essiam	OD− PIG +	73	2	75	42
		Denkyira	−OD PIG−	104	0	104	-
Volta	Keta	Anlo Afiadenyigba	+ OD PIG +	77	27	104	58
		Seva	+ OD PIG +	92	12	104	32
	North Tongu	Bla	+ OD PIG−	100	0	100	-
		Mepe	−OD PIG−	100	0	100	-
Eastern	Nsawam Adoagyire	Akwamu	OD + PIG−	100	0	100	-
		Dzatsui- Newtown	−OD PIG−	100	0	100	-
	Fanteakwa	Bepoase	−OD PIG +	81	21	102	63
		Ehiamankyene	OD + PIG +	59	4	63	25
Total				1365	105	1470	474

### Sampling technique

A purposive sampling technique was used to sample the study participants. Every male or female aged 1 and above and a resident of the study community was eligible to participate in the study. In each study community, pig farmers and community members were sampled representing occupationally exposed and unexposed populations respectively, while free-range and confined pigs were sampled. To ensure that as many as possible pig farmers in the study communities participated in the study, engagement with their local association and snowballing were used to recruit them.

### Data and sample collection and Processing

Blood samples (3–5 ml) from human participants and pigs were collected by trained phlebotomists and veterinarians respectively and allowed to clot. The samples were then centrifuged at 3000 rpm for 5 min for serum separation. The resulting serum samples were then transferred into labelled cryo-tubes and stored at −20 °C at the serology laboratory of the Noguchi Memorial Institute for Medical Research, University of Ghana.

Structured questionnaires containing closed and open ended-questions were administered to all consenting participants to obtain data on demographics, attitudes, and practices relating to sanitation water and hygiene, and contact with pigs and pork. This data was used to determine risk factors associated with HEV infection.

### Serological methods

All human serum samples were tested for anti-HEV antibodies Immunoglobulin G (IgG) (sensitivity 99.08%; specificity 99.90%) and Immunoglobulin (IgM) (sensitivity 97.7%; specificity 99.28%) using enzyme-linked immunosorbent assay (ELISA) and rapid immunochromatographic diagnostic kits (RDT) respectively. Pig samples were tested for anti-HEV total antibodies (Ab) using ELISA. Samples testing positive for any of these tests were then tested for HEV antigens (HEV-Ag) using ELISA. The HEV-Ag ELISA is an inexpensive alternative to PCR that can qualitatively detect HEV antigen in serum or plasma samples. The Wantai HEV-Ag ELISA (specificity 99.93%) detects the open reading frame 2 (ORF2) capsid antigen of HEV. All the diagnostic test kits were manufactured by Wantai Beijing Biopharmaceuticals, China. The assays from Wantai have been proven to have higher sensitivities than other commercial assays without loss of specificity [17–20]. Table 2 show the types of HEV markers and diagnostic test employed.

**Table 2:** HEV markers and test used

	HEV Marker	Test	Number of Samples Tested
Humans	IgM	RDT	1470 (All samples)
	IgG	ELISA	1470 (All Samples)
	Ag	ELISA	178 (IgM & IgG Positives)
Pigs	Ab	ELISA	474 (All Samples)
	Ag	ELISA	296 (Ab Positives)

### Data analysis

The data collected using the structured questionnaires and results of serological tests were entered into MS-Excel (2016). Statistical analysis was done using the Statistical Package for Social Sciences (SPSS) version 26.0 statistical software (IBM). Descriptive statistics of demographic parameters were computed and results presented as percentages. Binary logistic regression analysis was conducted in both univariate and multivariate models and odds ratios (OR) and their corresponding 95% confidence intervals (CI) used to determine risk factors associated with HEV infection. The Pearson chi-square (${x}^{2}$) test was used to test the association between categorical variables while significant difference was tested using a Z test. Significance was accepted at the level of *P* < 0.05.

## Results

### Demographic characteristics of study participants

Overall, 1470 participants were sampled from sixteen communities in four regions of Ghana. Of the total number of participants, 1365 (92.9%) were community members and 105 (7.1%) were pig farmers. A total of 877 of the participants were females (59.7%) and 593 (40.3%) males. The ages of the participants range from 1 to 92 years, with a mean age ± SD of 40.41 ± 22.24 years, a median of 40 years, and a mode of 60 years.

### HEV in humans

The overall seroprevalence indicating past or present exposure to HEV in humans was 12.6%.

This was higher in pig farmers (15.2%) than in community members (12.4%). However, the difference was not statistically significant (Z = 0.8506; *p* = 0.3953).

**Fig. 2:**
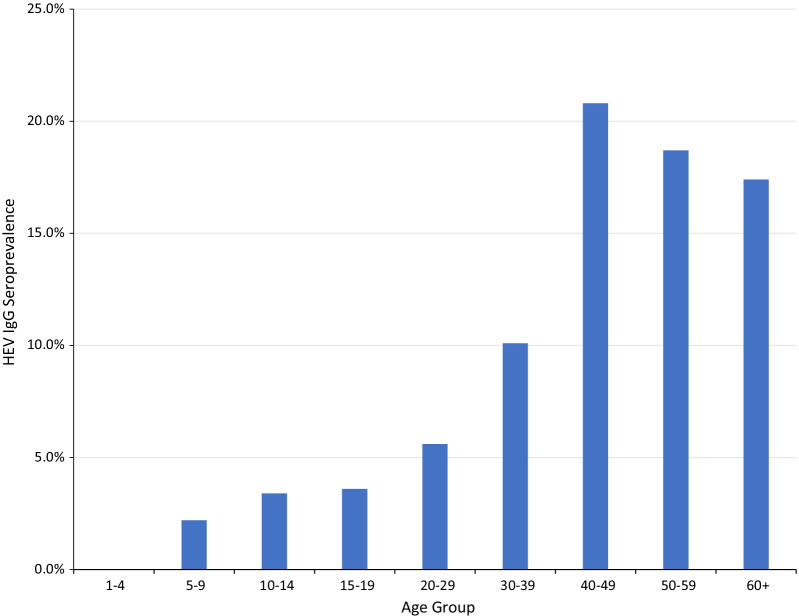
HEV IgG seroprevalence amongst age groups

**Fig. 3:**
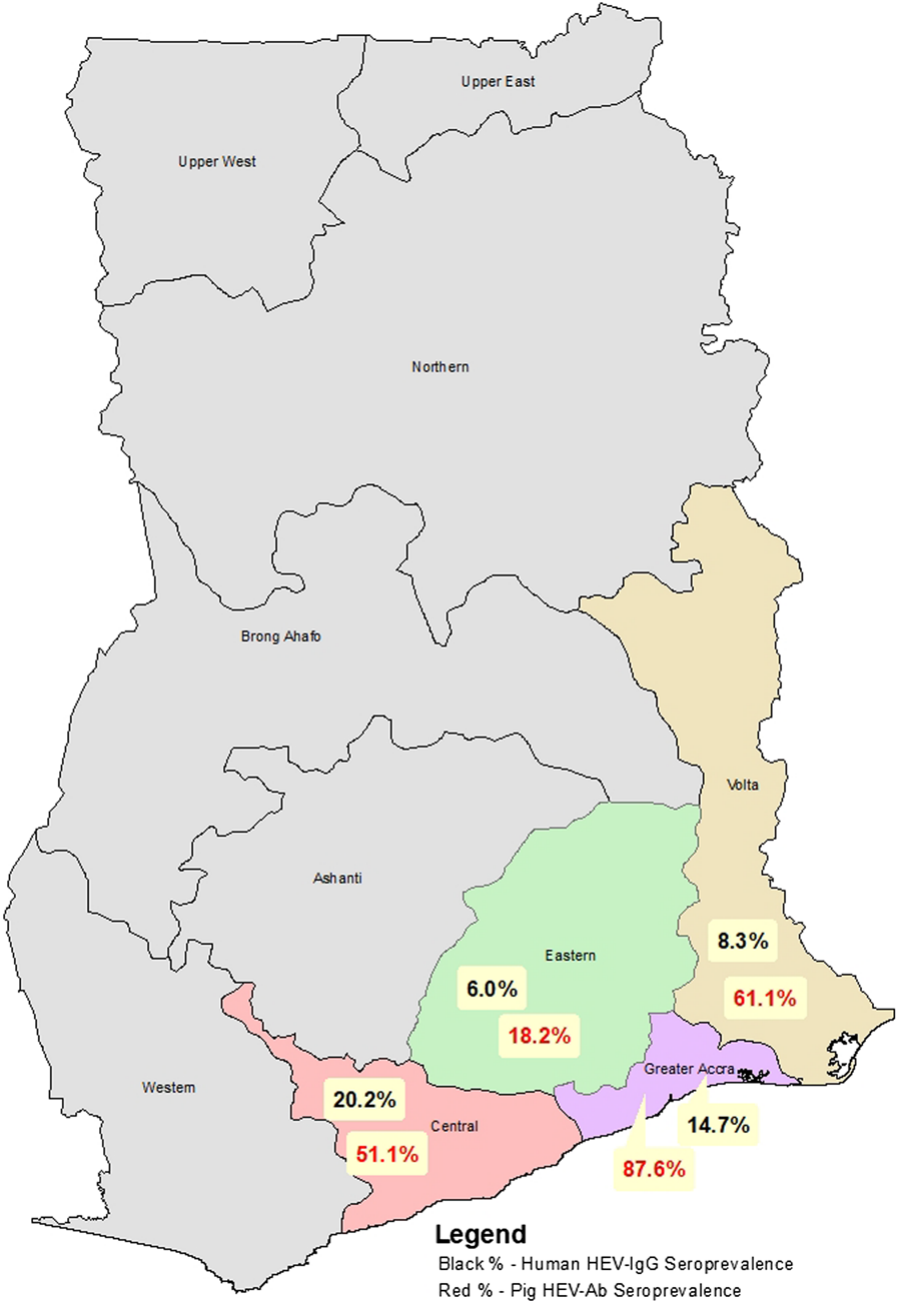
Regional distribution of HEV-Ab seroprevalence in humans and pigs

HEV IgG only seroprevalence in this study was 11.9%. HEV IgG seroprevalence was higher in males (13.3%) than females (11.3%), Table 3, but the difference was not statistically significant; Z = 1.173; *p* = 0.242. The HEV IgG seroprevalence was also not significantly different between community members (11.6%) and pig farmers (15.2%); (Z = 1.095; *p* = 0.2757). HEV IgG seroprevalence increased quite consistently and significantly with increasing age; ${x}^{2}$= 67.021; *p* = 0.000 as shown in Fig. 2. The HEV IgG seroprevalence was lowest (0%) in the 1–4 years age group and highest (20.8%) in the 40–49 years age group. Amongst the four regions, the HEV IgG seroprevalence ranged from 6.0% in the Eastern Region to 20.2% in the Central Region. It was significantly associated with region, ${x}^{2}$= 42.272; *p* < 0.00001. The HEV IgG seroprevalence in the Central Region (20.2%) and the Greater Accra Region (14.7%) were significantly higher than those in the Eastern Region (6.0%) and the Volta Region (8.3%). The regional distribution of HEV IgG seroprevalence in humans is shown in Fig. 3. Similarly, HEV IgG Seroprevalence significantly increased with the level of education as with age, with the lowest infections in persons with nursery education (1.7%) and the highest in persons with tertiary education (17.4%), (${x}^{2}$= 15.747; *p* = 0.008). HEV IgG seroprevalence amongst community types did not differ significantly; ${x}^{2}$= 4.485, *p* = 0.214; ranging from 9.9% in + OD PIG + communities to 13.9% in −OD PIG + communities.

**Table 3:** Demographics and HEV IgG seroprevalence

Demographic Variables	N	Percentage (Mean)	HEV IgG seroprevalence (%)	*P* Value
Gender				
Male	593	40.3%	13.3	0.242
Female	877	59.7%	11.3	
Age group				
1–4	44	3.0% (2.9)	0	
5–9	91	6.2% (7.0)	2.2	
10–14	87	5.9% (12.3)	3.4	
15–19	110	7.5% (17.1)	3.6	
20–29	196	13.3% (24.4)	5.6	0.000
30–39	199	13.5% (34.8)	10.1	
40–49	178	12.1% (44.3)	20.8	
50–59	209	14.2% (54.1)	18.7	
60 +	356	24.2% (69.7)	17.4	
Region				
Accra	341	23.2%	14.7	
Eastern	365	24.8%	6.0	
Volta	408	27.8%	8.3	0.000
Central	356	24.2%	20.2	
Educational level				
None	347	23.6%	16.7	
Nursery	59	4.0%	1.7	
Primary	346	23.5%	11.6	
JHS	552	37.6%	10.5	0.008
SHS	120	8.2%	10.8	
Tertiary	46	3.1%	17.4	
Population group				
Community Member	1365	92.9%	11.9	0.308
Pig Farmer	105	7.1%	15.2	
Community type				
−OD PIG−	304	20.7%	13.8	
−OD PIG +	261	17.8%	11.9	0.214
+ OD PIG−	388	26.4%	13.9	
+ OD PIG +	517	35.2%	9.9

HEV IgM only seroprevalence was 0.5%. Only community members tested positive for this antibody.

The prevalence (HEV-Ag) was 0.8%; It was significantly different in pig farmers and community members (2.9% vs 0.7%; Z = 2.412; p = 0.016). The seroprevalence and prevalence results are shown in Table 4.

**Table 4:** Seroprevalence and prevalence in community members and pig farmers

HEV test	Community member (n = 1365)	Pig FARMER (n = 105)	Total (n = 1470)	Total
Antibody				
IgM only	7 (0.51%)	0 (0%)	7	0.5%
IgG only	159 (11.6%)	16 (15.2%)	175	11.9%
IgM + IgG	3 (0.22%)	0 (0%)	3	0.2%
Overall Seroprevalence	**12.4%**	**15.2%**	**185**	**12.6%**
Antigen				
HEV Antigen	(9) **0.7%**	(3) **2.9%**	12	**0.8%**

## Demographic risk factors associated with HEV IgG *seroprevalence in humans*

A univariate logistic regression model showed that age group, level of education, and region were significant demographic risk factors for HEV IgG seropositivity; *P* = 0.0000, P = 0.0009, and *P* = 0.0000 respectively, Table 5. There was 1.4 times more risk of being seropositive for HEV for each age group from the lowest age group to the highest (OR 1.365; 95% CI 1.252—1.488). HEV IgG seroprevalence increased from 0.0% in the 1–4 years age group to 17.4% in the 60 + years age group. Similarly, there was 1.2 times more risk of being HEV seropositive associated with each level of education from the lowest to the highest. HEV IgG seroprevalence increased from 1.7% in persons with nursery education to 17.4% in persons with tertiary education. A 1.6 times more risk of HEV IgG seropositivity was associated with each region in this order; Eastern < Volta < Accra < Central. The risk factors for HEV IgG seroprevalence in humans are shown in Table 5.

**Table 5:** Logistic Regression of Demographic Risk factors for HEV IgG Infection in Humans

Variable	Coeff	StdErr	*P* Value	OR	(95% CI)		Overall Model Fit	
					Low	High	${x}^{2}$	*P*
Univariate logistic regression								
Gender	0.1888	0.1612	0.2415	1.2078	0.8806	1.6567	1.3635	0.2429
Age Group	0.3110	0.0440	0.0000	1.3648	1.2521	1.4876	61.5476	0.0000
Level of education	0.1648	0.0495	0.0009	1.1792	1.0701	1.2994	10.9089	0.0010
Category	0.2889	0.2841	0.3092	1.3350	0.7649	2.3300	0.9773	0.3229
Community type	− 0.1038	0.0693	0.1343	0.9014	0.7869	1.0326	2.2273	0.1356
Region	0.4786	0.0768	0.0000	1.6137	1.3883	1.8758	41.5532	0.0000
Multivariate logistic regression								
Gender	0.3496	0.1730	0.0433	1.4185	1.0106	1.9912		
Age Group	0.3144	0.0493	0.0000	1.3694	1.2433	1.5083		
Level of education	0.0454	0.0570	0.4254	1.0465	0.9359	1.1702	104.166	0.0000
Category	0.2857	0.2992	0.3397	1.3307	0.7403	2.3919		
Community type	− 0.0361	0.0733	0.6225	0.9645	0.8354	1.1136		
Region	0.4506	0.0777	0.0000	1.5693	1.3477	1.8272		

In a multivariate logistic regression model age group (*P* = 0.0000) and region (*P* = 0.0082) remained significant predictor of HEV IgG seropositivity along with gender (*P* = 0.0433). Males were 1.4 times more likely to be seropositive for HEV than females (OR 1.418; 95% CI 1.011—1.991). However, the HEV IgG seroprevalence in males and females was not significantly different (13.3% vs 11.3%; Z = 1.173, *P* = 0.242). Age group was significantly associated with HEV IgG seropositivity; the odds of HEV IgG seropositivity increased from 1.4 times in the 1–4 years age group to 16.9 times in the 60 + years age group. The region of residence as a demographic risk factor significantly predicted HEV IgG seropositivity with the highest risk in Central Region (OR 6.1), followed by the Greater Accra Region (OR 3.9) and the Volta Region (OR 2.5) with the lowest in Eastern Region (OR 1.7). Other demographic factors such as level of education, type of community, and population group did not show significant association with HEV IgG seroprevalence.

### HEV in pigs

A total of 474 pigs were sampled: 202 from the Greater Accra Region, 90 from Volta Region, 94 from Central Region, and 88 from Eastern Region. Free-range pigs were sampled from all four regions, whereas confined pigs were only available for sampling in the Greater Accra and Volta Regions. The sampled regions, number of pigs, and HEV-Ab seroprevalence as well as their *p* values are displayed in Table 6.

**Table 6:** Seroprevalence and prevalence in pigs

Region	Husbandry	Number	Seroprevalence	*P* Value	Prevalence	*P* Value
Accra	Confined	104	(93) 89.4%	0.424	(9) 8.7%	0.093
	Free-Range	98	(84) 85.7%		(3) 3.1%	
Volta	Confined	30	(2) 6.7%	< 0.00001	0	0.297
	Free-Range	60	(53) 88.3%		(4) 6.7%	
Central	Confined	0	0	-	–	
	Free-Range	94	(48) 51.1%		(9) 9.6%	–
Eastern	Confined	0	0	–	–	
	Free-range	88	(16) 18.2%		(1) 1.1%	–
Total	–	474	(296) 62.4%		(26) 5.5%	

An overall HEV-Ab seroprevalence of 62.4% was recorded in the pigs. HEV-Ab seroprevalences amongst the free-range pigs from the various regions were significantly different; ${x}^{2}$ = 113.4; *p* = 0.000. A significantly higher HEV-Ab seroprevalence was demonstrated in pigs in the Greater Accra Region (85.7%) and the Volta Region (88.3%) than in the Eastern Region (18.2%) and the Central Region (51.1%); P < 0.00001 amongst free-range pigs. Also, HEV-Ab seroprevalence in the Central Region was significantly higher than in the Volta Region (51.1% vs 18.2%; Z = 4.643, *P* < 0.00001). Amongst confined pigs, HEV-Ab seroprevalence was significantly higher in pigs in Accra (89.4%) than in pigs in the Volta Region (6.7%); Z = 8.791, *p* < 0.00001. The regional distribution of HEV-Ab seroprevalence in pigs is displayed in Fig. 3. Amongst the regions, HEV-Ab seroprevalence between confined and free-range pigs was significantly different only in the Volta Region; 6.7% vs 88.3%; Z = 7.492; *p* < 0.00001, Table 6.

An HEV prevalence (HEV-Ag) of 5.5% was recorded in the pigs. The prevalence amongst both confined and free-range pigs ranged from 1.1% in the Eastern Region to 9.6% in the Central Region. Prevalence between confined (8.7%) and free-range (3.1%) pigs in Accra was not significantly different; (Z = 1.6806, *P* = 0.09296).

### Risk factors of HEV-Ab seroprevalence in pigs

HEV-Ab seroprevalence showed a significant association with husbandry and region for pigs in Accra and Volta region in both univariate and multivariate logistic regression models, Table 7. A significantly higher HEV-Ab seroprevalence was found in free-range pigs compared with confined pigs (86.7% vs 70.9%); Z = 3.333; *p* = 0.0086. The odds of HEV-Ab seroprevalence amongst free-range pigs was seven-fold higher than in confined pigs in the multivariate model (*p* = 0.0000). Also, the odds of HEV-Ab seroprevalence was about 5times higher for pigs in Accra than pigs in the Volta Region (*p* = 0.0000).

**Table 7:** Seroprevalence, prevalence and risk factors in pigs

		Univariate				Multivariate			
Husbandry (Accra and Volta)	HEV Ab	OR	Low	High	*P* value	OR	Low	High	*P* value
Confined	95 (70.9%)								
Free-range	137 (86.7%)	2.6782	1.4823	4.8388	0.0011	7.0506	3.5580	13.9716	0.0000
Region									
Accra	177 (87.6%)								
Volta	55 (61.1%)	4.5055	2.4831	8.1750	0.0000	4.6015	2.3003	9.2048	0.0000

Husbandry (Accra and Volta)	HEV-Ag	OR	Low	High	*P* value	OR	Low	High	*P* value
Confined	9 (6.7%)								
Free-range	7 (4.4%)	0.6439	0.2332	1.7780	0.3956	0.6663	0.2380	1.8653	0.4396
Region									
Accra	12 (9.0%)								
Volta	4 (2.5%))	1.3579	0.4257	4.3312	0.6052	1.2605	0.3888	4.0867	0.6997

Husbandry and region, however, were not significant predictors of HEV-Ag prevalence in both univariate and multivariate logistic regression models. Table 7 shows the univariate and multivariate logistic regression of risk factors associated with HEV seroprevalence and prevalence in pigs.

## Discussion

### HEV in humans

In this study, seroprevalence, prevalence and risk factors for human and pig infection with HEV were explored to determine the burden of the disease and the contribution of zoonotic and WASH-related transmission routes in Ghana.

The results show an overall seroprevalence of 12.6% and prevalence of 0.8% in humans in Ghana. This prevalence indicates endemic circulation of HEV in the study communities and Ghana at large which warrants action. There was no significant difference in overall seroprevalence between pig farmers and the general public in this study. The overall seroprevalence of 12.4% in community members in this study was lower than the 13.4% previously reported in blood donors by Meldal et al., [12] in Ghana. The difference in seroprevalence between the two studies could be because this study covered a much broader population, age range, and regions in Ghana than the other. The effect of differences in time and diagnostics assays used could also be significant factors. Also, the overall seroprevalence in community members in this study was much lower than the seroprevalence of 47.9% recorded in healthy people in Nigeria [21]. The difference in seroprevalence between these two studies may be reflective of variation in sample size and age range of research participants. Moreover, differences in sanitation practices, socioeconomic status, and level of exposure of participants to risk factors of HEV infection could be possible reasons. In Asia, an HEV seroprevalence of 11% each was reported in healthy people in Taiwan [22] and Mongolia [23] which are very close to the seroprevalence in this study.

HEV seroprevalence of 15.2% (IgG) and 0% (IgM) was recorded for pig farmers in this study compared with 0% HEV IgG and the 38.1% HEV IgM seroprevalence reported by Adjei, Aviyase [24] in pig handlers in Ghana. It is unclear why Adjei did not record any HEV IgG but a high HEV IgM. Exposure of an HEV naïve population to infections for the first time could be the reason. As most of the participants had been working on the pig farms for less than a year and HEV infection was significantly associated with persons who had been working on the farm for less than one year.

Compared with other serosurveys in Africa, HEV seroprevalence in pig farmers in this study was considerably lower than the 58.3% and 76% recorded in animal handlers in Nigeria and butchers in Burkina Faso respectively [21, 25]. However, it is comparable with the HEV seroprevalence of 14.1% reported in pig butchers in Madagascar [26]. The dissimilarities in HEV seroprevalence between occupationally at-risk persons in these studies may be influenced by the level of exposure of pig farmers to HEV-infected pigs and the level of prevalence of other predisposing factors for HEV transmission.

HEV prevalence in this study was low, as revealed by the overall HEV antigen (HEV-Ag) prevalence of 0.8% which is supported by the very low recent infection (IgM) of 0.5%. This could be characteristic of a low level of HEV transmission rate.

### Demographic risk factors associated with HEV IgG seroprevalence in humans

Demographic factors were explored in both the univariate and multivariate models to determine risk factors associated with HEV seropositivity. While age group, level of education, and region were associated with HEV seropositivity in the univariate analysis, gender, age group and region were significant predictors of HEV in the multivariate analysis.

Age group significantly predicted HEV IgG seropositivity; the trend of increasing HEV IgG seroprevalence with increasing age in this study is consistent with many reports of HEV studies across the world; in India [27], Kenya [28], CAR [29], Indonesia [30], Taiwan [22], Spain [31], USA [32], and Germany [33]. Since HEV IgG is a marker of exposure and persists for long periods (up to 14 years) [34], the HEV IgG seroprevalence is bound to be higher in older than in younger individuals. Thus, HEV IgG seroprevalence increases with age as a consequence of accumulated infections over time.

While the risk of HEV infection was associated with increasing education this observation is probably influenced by age since higher education level correlates with increasing age. This can be clearly inferred from the similarity in HEV IgG seroprevalence amongst the tertiary and none educated groups. The HEV IgG seroprevalence in persons without education was similar to those who had tertiary education, 17.4% vs 16.7%. All persons who were positive for HEV IgG seroprevalence in the none educated category were adults aged 20 years and above; education level appears to be a confounder.

Gender was significantly associated with HEV IgG seroprevalence in the multivariate analysis with a higher risk in males than in females. However, the odds ratio was not very high (1.4) and the difference in HEV IgG seroprevalence was not statistically significant, Z = 1.173; *p* = 0.242.

HEV IgG seroprevalence was significantly associated with the region of residence. The study communities in the Central and Greater Accra regions where HEV IgG seroprevalences were higher were more urban than communities in the Volta and Eastern region where HEV IgG seroprevalences were low. Many studies have found higher HEV IgG seroprevalence in rural than in urban areas [14, 21, 35, 36]. In this study, however, it seems to be associated with increasing urbanisation as reported in studies in Gabon [37] and India [38]. Overcrowding in urban areas puts pressure on sanitation and water facilities, creating unhygienic conditions which promote WASH-related HEV transmission. Such conditions are more prevalent in the Greater Accra and Central region than in the Eastern and Volta regions.

### HEV in pigs

Several serosurveys from many countries across the world have shown high HEV seroprevalence in domestic pigs with increased risk of infection in humans through direct contact and consumption of undercooked infected pork products [39]. Unfortunately, studies investigating HEV seroprevalence in domestic pigs and the level of risk of zoonotic transmission from pigs to humans are lacking in Ghana. This study is amongst the few studies jointly investigating HEV in humans and pigs in Ghana.

The high HEV-Ab seroprevalence of 62.4% demonstrates clearly that HEV is endemic in pig populations in Ghana. The widespread practice of free-range pig husbandry in major pig rearing communities in Ghana exposes pigs to the risk of environmental HEV infections. Also, the practice of introducing free-range breeding stock to intensive farms introduces the disease to these systems.

Only a few HEV seroprevalence studies of pigs are available in Ghana for comparison with the results from this study. The results support the high HEV seroprevalence of 85% in our previous study in pigs from the Greater Accra and Upper East regions of Ghana. These results altogether enable us to gauge the national HEV seroprevalence as well as the regional distribution of HEV seroprevalence in pigs in Ghana. The HEV-Ab seroprevalence of 62.4% in this study is lower than the 85% found in our previous serosurvey [16], and the 77.5% reported by El-Duah, Dei [40] in the Ashanti region of Ghana**.** However, the HEV-Ab seroprevalence of 87.6% in this study and the 80% in our previous study both in the Greater Accra region are comparable.

When compared with HEV seroprevalence values in pigs from other African countries, our findings were comparable with results in Madagascar [26] and Nigeria [41]**.**

HEV antigen prevalence was relatively low in pigs in Ghana at 5.5% compared with the 64.2% seropositive for HEV antibodies. Other studies of HEV prevalence have used PCR on liver samples and so cannot be compared to this study which used antigen ELISA on serum samples. These studies recorded HEV RNA prevalence of 0.9–10.1% [26, 40, 42, 43].

## Conclusion

This study explored the prevalence and seroprevalence of HEV and risk factors for infection in humans and pigs in Ghana. The results show that HEV infection is endemic in both occupationally at-risk persons and the general population in Ghana. Overall seroprevalence (12.6%) was much higher than HEV prevalence (0.8%).

The results also show that HEV is endemic in domestic pig populations which serve as an HEV reservoir. The pervasive free-range pig production system predisposes roaming pigs to environmental HEV. Likewise, scavenging pigs can contaminate the environment by shedding the virus in faeces and urine. It is therefore important that authorities enforce the laws proscribing free-range pig production to prevent this and reduce the risk of HEV infection in pigs. Overall, this study helps define the burden of HEV in human and pig populations in Ghana. Although the HEV prevalence seems to be minimal, the overall seroprevalence is a cause for concern.

Due to low participant responses, the desired sample sizes could not be achieved in some communities. Also, a molecular detection method (RT-PCR) could not be employed in this study due to financial and logistical constraints. These challenges could constitute a limitation of this study. Isolation and characterisation of HEV in humans, animals, and water simultaneously will be key in determining the main transmission route of HEV in Ghana. This notwithstanding, the study brings to light the problem of HEV in Ghana.

## Data Availability

The datasets used and/or analysed during the current study are available from the corresponding author on reasonable request.

## Abbreviations

Ab: Antibody
CI: Confidence interval
ELISA: Enzyme-linked immunosorbent assay
HEV: Hepatitis E virus
IgG: Immunoglobulin G
IgM: Immunoglobulin M
OD: Open defaecation
OR: Odds ratio
PCR: Polymerase chain reaction
RDT: Rapid Immunochromatographic diagnostic
RNA: Ribonucleic acid
SPSS: Statistical package for social sciences
WASH: Water, sanitation, and hygiene
